# Measurement of lipid flux to advance translational research: evolution of classic methods to the future of precision health

**DOI:** 10.1038/s12276-022-00838-5

**Published:** 2022-09-08

**Authors:** Amadeo F. Salvador, Chi-Ren Shyu, Elizabeth J. Parks

**Affiliations:** 1 Department of Nutrition and Exercise Physiology, University of Missouri, Columbia, MO 65212 USA; 2 Department of Medicine, Division of Gastroenterology and Hepatology, School of Medicine, University of Missouri, Columbia, MO 65212 USA; 3 Department of Electrical Engineering and Computer Science, Institute for Data Science and Informatics, University of Missouri, Columbia, MO 65211 USA

**Keywords:** Metabolomics, Translational research, Machine learning

## Abstract

Over the past 70 years, the study of lipid metabolism has led to important discoveries in identifying the underlying mechanisms of chronic diseases. Advances in the use of stable isotopes and mass spectrometry in humans have expanded our knowledge of target molecules that contribute to pathologies and lipid metabolic pathways. These advances have been leveraged within two research paths, leading to the ability (1) to quantitate lipid flux to understand the fundamentals of human physiology and pathology and (2) to perform untargeted analyses of human blood and tissues derived from a single timepoint to identify lipidomic patterns that predict disease. This review describes the physiological and analytical parameters that influence these measurements and how these issues will propel the coming together of the two fields of metabolic tracing and lipidomics. The potential of data science to advance these fields is also discussed. Future developments are needed to increase the precision of lipid measurements in human samples, leading to discoveries in how individuals vary in their production, storage, and use of lipids. New techniques are critical to support clinical strategies to prevent disease and to identify mechanisms by which treatments confer health benefits with the overall goal of reducing the burden of human disease.

## Lipid metabolism: tracking lipid and precision medicine

Personalized tracking of how lipid (fat) metabolism changes over time could lead to improvements in the diagnosis and treatment of several diseases. Elizabeth Parks and colleagues from the University of Missouri, Columbia, USA, discuss the ways in which researchers use stable isotope labeling to monitor the kinetics of fatty acids and other lipids in the body. Usually, lipid quantities are measured only at a single timepoint, however the tracking of lipid turnover over time provides further diagnostic information. Aided by new techniques such as high-throughput mass spectrometry and machine learning, researchers are now able to continuously map total lipid contents in individual patients. The transition of measurements of lipid flux from the research laboratory to the doctor’s office will likely play a role in a new era of precision medicine.

## Introduction

Currently, in the field of medicine, for most biomarker analysis, a single blood draw is performed with the patient in a fasted state so that a standardized set of analytes may be measured and used to monitor the patient’s health status. For lipids, this protocol includes the measurements of plasma total cholesterol (TC), triacylglycerols (TG), and high-density lipoprotein cholesterol (HDLc), with either the calculation or the direct measurement of low-density cholesterol (LDLc). This analysis strategy has developed over the past 60 years based on very large cohorts of individuals studied prospectively (e.g., the Framingham study), and the data have provided a strong basis for prediction of disease risk and lipid monitoring to clinically manage disease outcome. However, in contrast to a fasting concentration of an analyte in plasma, the biological processes that directly lead to disease development include the production and catabolic rates of macromolecules (and micromolecules)^1^. For lipids, parameters such as the turnover rate of very low-density lipoprotein (VLDL)-TG are correlated with a molecule’s concentration in blood^2^. This type of analysis suggests that data from kinetic parameters may be better predictors of disease^1^. The goal of the present review is to describe the evolution of kinetic measurements in the field of lipid metabolism. We discuss how the field’s targeting of metabolite flux measurements, based on isotope turnover, is contrasted with the rapidly advancing technologies that allow untargeted analysis of hundreds of molecules simultaneously. The review concludes with a description of the challenges of combining lipidomics with isotopic tracing and highlights the promise of this field in making discoveries in precision health through the use of artificial intelligence and machine learning.

## Evolution of lipid metabolism knowledge

**Fig. 1:**
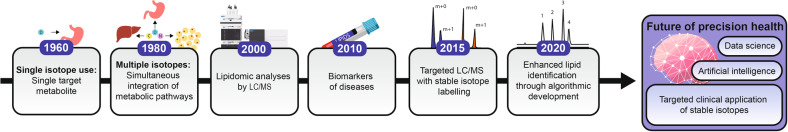
Development of techniques used to investigate lipid synthesis, the identification of the lipidome and the integration of data science. The figure describes the evolution of the study of lipid metabolism––from the use of isotope tracers to the development of lipidomics––toward the promise of precision health studies using artificial intelligence and machine learning.

In the body, the synthesis, transport, and degradation of lipids shifts throughout the day and depends on the organism’s energy status (e.g., plane of nutrition, including feeding, fasting, exercising, and starvation). Among their important functions, lipids are produced to support membrane synthesis and to serve as precursors of other key molecules, such as steroid hormones, and as major contributors to energy production through beta-oxidation. Knowledge of lipid synthesis and TG storage is critical to understanding the causes and consequences of obesity and the relationship between excess body weight and chronic disease risk. Research on cholesterol synthesis and lipoprotein transport has provided the basis for understanding the causes of atherosclerosis and the development of treatments for cardiovascular disease^3^. Measurements of lipid production and turnover are possible through the use of radioactive and stable isotopes, which can be fed to or infused into humans, and then samples of blood or tissue are collected^4^. The evolution of this field is shown in Fig. 1. The early understanding of cholesterol and TG metabolism in humans came from elegant studies and mathematical modeling led by scientists including Mones Berman, Robert Levy, Robert Phair, Scott Grundy, and Barbara V. Howard, among others, and this field began with the utilization of radioactive isotopes^5,6^. For example, from the 1980s to the 1990s, studies utilized the infusion of a glycerol tracer to quantitate TG kinetics^7^. Such seminal studies of lipid metabolism traced both the lipids carried in lipoproteins and the apolipoproteins on the surface of the particles^8^. Amino acid tracers were used to label apolipoproteins, and the importance of these methods extends right up to the present day, as recently reviewed by Ying and colleagues, to understand how variations in two genes, *proprotein convertase subtilisin/kexin type 9 (PCSK9)* and *angiopoietin-like 3 (ANGPTL3)*, impact concentrations of plasma LDLc^9^. As shown in Fig. 1, in the years just before and after 2000, advancements in mass spectrometry led to the ability to measure multiple lipids simultaneously, and the Lipid Maps Consortium was established in 2003. As described below, Lipid Maps provided the essential standardization of lipid nomenclature and protocols, which has supported the large gains made in the field of lipidomics over the past 20 years.

### General considerations in working with lipid tracers

Numerous excellent reviews exist on the use of isotope tracers to understand lipid metabolism^9–13^. Unlike the study of carbohydrate metabolism, where tracing metabolism primarily requires the infusion of labeled glucose (because glucose is the dominant molecule used for energy generation), the study of lipid metabolism using isotopes is complicated by a number of factors (Table 1). These include the various species of lipids present in the body (TG, cholesterol, phospholipids); the different molecular structures of these species; and within fats that contain acyl chains, the fact that fatty acids vary in chain length, saturation, and structure (presence or absence of N or oxygen). Thus, to investigate the production of a given lipid species, a single component of the molecule is typically chosen for administration as a label. For example, the TG production rate by the liver can be labeled by bolus injection or infusing either a tracer of glycerol (d_5_-glycerol) to label the backbone of TG or a tracer of fatty acid (^13^C_4_-palmitate) to label the acyl chain^14^. However, different fatty acids are handled differently in the body such that the choice of which labeled fatty acid to administer depends on the research question. For example, if the research pertains to the rate of biological fatty acid oxidation, long-chain saturated fatty acids (SatFat) are less oxidizable than monounsaturated fatty acids (MUFA) and polyunsaturated fatty acids (PUFA)^15^. For measurements of whole-body fatty acid oxidation, both the SatFat, palmitate^16^, and the MUFA, oleate, have been chosen^17^, and the differences in the body’s handling of these two fatty acids need to be taken into consideration when interpreting the results. Some investigators have simultaneously administered labeled SatFat, MUFA, and PUFA in a single experiment^18,19^ to assess global fatty acid oxidation irrespective of fatty acid saturation. Methodological aspects have been investigated^20^, and these methods have been used to understand how the quantity of fat in the diet influences body weight and energy balance.

**Table 1:** Analytical factors affecting lipid analysis.

Different fates of fatty acids with different chemical structures: Metabolic flux is dependent on a fatty acid’s characteristics. For example, the oxidation of saturated fatty acids decreases with increasing carbon length.
Different lipid species: Lipidome diversity constrains the ability to analyze several lipids using a single methodology. This also increases *m/z* ratio overlap in chromatograms. Different lipids require different separation techniques.
Synthesis interrelationships: Increases in the synthesis of one lipid species may affect the synthesis of a related species, resulting in complex patterns of isotopic labeling.
Choice of internal standard: Different internal standards are needed when lipids are labeled at multiple sites.
Biological matrices: Lipids can be found in different tissues and associated with different lipoproteins (e.g., VLDL, IDL, LDL, and HDL).
Lipid aggregation: At high concentrations, lipids are likely to form aggregates (e.g., dimers, oligomers, or micelles), hindering lipid detection by mass spectrometry.
Low abundance: Lipids may serve as intermediates and signaling molecules present in low abundance in biological systems.
Isotopic labeling protocols: Different molecules and pathways will require different protocols.

*m/z* mass divided by charge, *VLDLs* very low-density lipoproteins, *IDL* intermediate-density lipoproteins, *LDL* low-density lipoproteins, *HDL* high-density lipoproteins.

### Tracing the entry of dietary fats and their fates

Many methods are available to assess meal lipid absorption and the fate of dietary fatty acids. Labeling with SatFat^21,22^, MUFA^23,24^, or PUFA^25^ can be used. Feeding a meal containing a labeled lipid and measuring the appearance of the lipid in blood to track intestinal chylomicron production rate has added important data to understand the phenomenon termed the “second meal effect,” which describes the intestine’s release of lipids from an earlier meal that were stored in the cytosol of the enterocytes^26^. Previously consumed lipids are released into the blood at the very onset of the next episode of eating, and such data have supported the recent development of a mathematical model^27^ suggesting that (1) the intestine possesses the ability to store a large amount of lipids between meals and (2) this storage and subsequent release is a hallmark of insulin sensitivity. Our group observed less intestinal storage in individuals who were insulin resistant. Furthermore, as dietary isotopes of fat were added to meals, it became clear that meal lipids carried in chylomicrons can contribute to the plasma FFA pool through intravascular lipolysis by lipoprotein lipase (LPL). This phenomenon, termed “spillover,” describes the appearance of dietary fatty acids through an increase in plasma FFA after meals^28^. For some researchers unfamiliar with the spillover process, it is assumed that in a fasted state, the plasma FFA pool emanates solely from adipose tissue lipolysis. However, when humans^21,29^ or mice are fed high-fat diets, they exhibit higher blood TG concentrations^30^, and fatty acids can be liberated from TG-rich lipoproteins via the action of LPL at a rate that exceeds tissue uptake. In the plasma compartment, the fatty acids are carried on albumin. After the consumption of a mixed meal, insulin suppresses the rate of adipose fatty acid release, lowering the plasma FFA concentration, while at the same time, the process of spillover can replace some of those fatty acids, increasing the FFA concentration^22^. The higher the meal-TG content is, the greater the chylomicron TG concentration and the greater the spillover, resulting in an underestimation of adipose lipolysis suppression by insulin and the potential erroneous conclusion of apparent adipose insulin resistance. When one fatty acid isotope is added to the meal and a different isotope is infused directly into the plasma FFA pool, the absolute rate of spillover can be quantitated. This method has been published only once^2^, and in that study, the measurements were made after subjects had not eaten for 16 h. Residual spillover from the previous evening meal’s TG was small when measured in a fasted state^2^, while the spillover effect was largest directly after meals^29^. Future studies should determine how the process of spillover leads to dietary fat contribution to ectopic lipid deposition.

### Multiple-isotope labeling of a lipid pool

The methods described above have been used to trace the synthesis and biological fates of a lipid species by infusing a single labeled molecule. In contrast, our laboratory has developed a multiple-isotope tracing protocol to quantitate the various sources of fatty acids used for liver lipid synthesis^31^. Liver-TG can be synthesized from four pathways: (1) those originating in the diet and cleared to the liver, (2) plasma that originate in adipose tissue, (3) fatty acids prestored in hepatocytes, and (4) liver fatty acids that have been newly synthesized from carbohydrates and other precursors through the pathway of de novo lipogenesis (DNL). Although SatFat, MUFA, and PUFA are all used for liver-TG synthesis, because they are oxidized in organs at different rates, for investigations of the contribution of the four different pathways, one type of fatty acid (a saturated fatty acid or a monosaturated fatty acid) is used to trace all the pathways simultaneously. Because palmitate is the primary product of DNL, we chose palmitate as the tracer for all biochemical pathways. In this way, ^13^C_1_ acetate might be infused to label the DNL pathway found in newly made palmitate, while at the same time, ^13^C_4_ palmitate is infused, and a per-deuterated TG with palmitate at all three positions is fed in a meal. During isotopic administration, blood is drawn, VLDL-TG are isolated, and TG palmitate is analyzed by GC/MS to look for the various isotopomers that represent the different pathways of palmitate flux into TG. Specific to this example, an M1 and M2 palmitate would be used to trace DNL, an M4 palmitate would be used to trace the contribution of the plasma FFA pool to liver-TG synthesis, and an M31 palmitate would be used to detect the recycling of dietary fatty acids into liver-TG stores. Methods have been established for multiple isotopic labeling^32^ that can be accomplished in humans, rodents^33,34^, and primates^35^.

For isotope tracer methods measuring in vivo metabolism that require IV infusion preparation, multiple cannulations are needed for IV infusion of the isotope infusion and blood sampling, and samples may be collected through the breath or through tissue biopsies. These procedures require long visits to the clinic site with the participant connected to an infusion pump and thus restrict the experiment to only an acute measurement period (e.g., 3–24 h). These limitations of traditional isotope IV infusions can be mitigated by utilizing the less invasive methodology of oral ingestion of isotope tracers such as acetate^36^ or deuterated water (d_2_O)^37^. The mention of d_2_O first occurred in the 1930s in a 19-day animal study where fatty acid synthesis and “destruction” (as mentioned by the author) were determined^38^. In today’s studies, d_2_O is provided for oral consumption on an outpatient basis, and in the area of lipid metabolism, oral administration of heavy water has been used to assess fatty acid synthesis^2^, cholesterol^39^, ceramide^40^, TG^41^, and apolipoprotein synthesis^42^. As described next, heavy water labeling is a powerful tool, and recent developments have uncovered analytical considerations that need to be considered when using this tracer.

## Research needs to support the integration of isotopes in precision medicine

### High-throughput mass spectrometry and the promise of fluxomics

As described above, the understanding of how to use isotopes to quantify lipid metabolism has been well-established for decades, and developing alongside this field, extremely quickly, is the ability to map the entire lipidome (Fig. 1). The current challenge is to bring these two fields together to understand the interrelationships between lipid fluxes, how different synthetic rates lead to elevations or reductions in concentrations, and how these fluxes contribute to disease development differently between individuals. To accomplish this, isotope tracing in lipidomic studies (so-called fluxomics) has begun, with demonstrated success in in vitro studies^43^. Of the many technical challenges that have been identified, long-term labeling with d_2_O results in shifts in the retention times and m/z of many molecules at once, producing species that overlap with naturally occurring molecules. These new patterns of spectra can also disrupt the efficiency of automated peak identification programs. As recently highlighted by Downes et al., some challenges can be overcome by isotope fragmentation, increased sampling density^44^, and the use of high-resolution MS^45–47^. Attention should be given to the choice of internal standards^48^ (which are typically deuterated themselves), and efforts are underway to expand methods for the development of lipidomic standards^49^. A comprehensive paper on the accurate quantitation of lipids has been published by Han and colleagues^50^, while Satapati et al. recently reviewed the advantages of isotope-labeled flux measurements in preclinical studies, with particular emphasis on lipid tracing^51^. Continued advances in analytical capabilities will support the development of precision medicine strategies, which is currently one focus of the NIH (https://acd.od.nih.gov/working-groups/pmi.html).

### Technical advancements to facilitate the use of isotopes in human research

Challenges in flux measurements include the extensive time between sample collection and analysis and the lack of ease with which the equipment can be used. One strategy might be to send samples from a medical facility to a centralized core for mass spectrometry analyses. Alternatively, as automated methods advance, isotope analysis may become available for on-sight use. Three new developments occurring in other fields are examples of technical events that may aid research in fluxomics in the future. First, rapid evaporative ionization mass spectrometry (REIMS) is a technique that generates aerosols during surgery^52^. The molecular ions present in the aerosols of a specific tissue are analyzed by mass spectrometry to immediately determine tissue composition^53^. A similar technique, called the MassSpec Pen, provides a probe that utilizes on-site sampling of tissue profiling that maintains tissue integrity^54^. If these mass spectrometry-based technologies are extended to the detection of heavier isotopes, they can be used to detect tissue-specific labeling patterns without the need for the conventional time it takes for sampling and analysis. Second, recent advances in miniaturization and portability of mass spectrometers will lead to increased use within a wider arena of science and potentially their use in medical facilities^55^. Third, over the past decade, advancements in ‘smart pumps’^56^ and other wearables have provided the potential to expand beyond quantifications of analyte concentrations to include measurements of mass spectra, thus enabling flux to be measured on an outpatient basis. Increasing the usability of mass spectrometry will support the study of larger cohorts of people in precision medicine research. In addition to these technical developments, the field will need to expand the workforce for the analysis of the data and multidisciplinary approaches, including data science and artificial intelligence (AI).

### Data science, artificial intelligence, and machine learning

Two computer-based techniques that will significantly influence the future of lipid research are artificial intelligence (AI), which enables computers to mimic the human decision-making process, and machine learning (ML), in which data analysis includes automated, analytical model building. These techniques have already improved mass spectrometry software through the detection of chromatographic peaks by improvements in noise filtration^57^ and have been extended to the field of medicine in which ML of lipidomic data is proposed to improve the diagnosis of fatty liver disease^58^. A next step in this field will include the integration of lipidomics with genetic information. In the area of lipid metabolism, much is currently known about the influence of single gene variants on increasing human disease risk (e.g.^59^), but no studies to date have combined fluxomic and broader genetic analyses. Recently, a mouse genome/lipid association study identified genetic regulators of lipid species^60^. The authors created, validated, and provided free access to LipidGenie (http://lipidgenie.com), a web-based resource that allows researchers to evaluate relationships between lipidomic and genomic data. Although single gene variants that influence lipid metabolism are known in humans (e.g.^61^), extending this work to understand lipid flux against a background of human genetics will be possible only with AI. In addition, AI empowers the analysis of not only “long data,” where the number of is greater than the number of input variables, but also “wide data,” where the number of variables is greater than the number of subjects^62^. A wide dataset is a common characteristic of both tracer and lipidomic studies, demonstrating the importance for lipid researchers to be introduced to AI concepts. Human metabolic flux studies are also characterized by high interindividual variation^23^, another scenario where AI can be beneficial. Through the lens of AI, Berry et al. successfully predicted postprandial TG and glucose concentrations, and the ML model was able to associate postprandial response to cardiovascular disease risk^63^. With the advance of the 2020–2030 strategic plan for nutrition research created by the NIH^64^, large-scale, open-source datasets will be available, encouraging the collaboration of AI and metabolism researchers. While data modality and volume are rapidly increasing, innovative explainable AI methods will become more important for automatic cohort stratification to tailor interventions for targeting patients^65,66^. Deeper phenotypic characterization of individuals will be essential for personalized treatments. The better connection between fluxomics and AI will not only improve lipid tracer protocols but also provide a possible bridge through which tracers will support precision medicine initiatives.

### The extended vision of isotopic analysis in medicine

Although the time needed to analyze isotopic data is being reduced, no current initiative exists to link the fluxes measured by tracers to medical diagnoses, with the potential exception of the field of cancer, in which metabolic phenotyping of tumors is on the horizon^67^. However, the evolution from the benchtop to the doctor’s office will not happen without the fundamental infrastructure created by precision medicine studies. Several approaches have been proposed to integrate isotopic technologies with medical procedures. Beysen et al.^68^ added labeled glucose to create the deuterated-glucose disposal test (^2^H-GDT). The ^2^H-GDT quantitates whole-body glycolysis in humans, resulting in data that are strongly correlated with glucose disposal estimated from a euglycemic-hyperinsulinemic clamp. Along the same line, Behn et al. added a glycerol tracer to an oral glucose tolerance test, followed by NMR analysis with the goal of developing an oral minimal model^69^. The one-compartment model accurately simulated glycerol concentrations and predicted postprandial adipose lipolysis. These types of initiatives increase the applicability of tracer methodology since oral glucose tolerance tests are frequently employed in medical practice. It is striking that current recommendations for pharmacotherapy are based on common national guidelines for the treatment of patients at risk (statin treatment, for example), yet the question arises as to how such guidelines will change as precision medicine strategies evolve. Recent efforts to expand precision medicine research will support the development of future techniques such as those described above that harness the potential of flux measurements to improve individualized disease diagnosis and treatment.

## Conclusions

Stable and radioactive isotopic techniques have provided fundamental information on the production and turnover of lipids in the circulation and within organs. The knowledge obtained from these studies advanced the understanding of the etiology of atherogenic dyslipidemias and uncovered the mechanisms by which lipid-lowering therapies provide health benefits. Over the years, techniques for tracing the synthesis and fates of lipids have been established, beginning with the use of single-isotope tracers and evolving to use multiple tracers simultaneously. Technical advances in the field of lipidomics have outpaced the use of isotopic techniques, and investigators are currently exploring ways to combine these two fields. In contrast, precision medicine researchers seek to understand how patients may respond differently to pharmacotherapy and lifestyle changes. Data science and AI are absolute requirements to be able to leverage large-scale studies using fluxomics, and the emerging ML technologies could personalize medicine through the wide output of data with less need for sampling. Through team science, techniques and procedures will be created that will shape the future of discoveries in lipid biology and medical care.
